# The involvement of insulin-like growth factor 2 binding protein 3 (IMP3) in pancreatic cancer cell migration, invasion, and adhesion

**DOI:** 10.1186/s12885-015-1251-8

**Published:** 2015-04-11

**Authors:** Clarissa C Pasiliao, Che-Wei A Chang, Brent W Sutherland, Shannon M Valdez, David Schaeffer, Donald T Yapp, Sylvia S W Ng

**Affiliations:** 1 Department of Experimental Therapeutics, British Columbia Cancer Agency, 675 West 10th Avenue, Vancouver, BC V5Z 1 L3 Canada; 2 Department of Pathology and Laboratory Medicine, Faculty of Medicine, University of British Columbia, Vancouver, BC V6T 2B5 Canada; 3 The Pancreas Centre BC, 2775 Laurel St., Vancouver, BC V5Z 1M9 Canada; 4 Faculty of Pharmaceutical Sciences, University of British Columbia, Vancouver, BC V6T 1Z3 Canada

**Keywords:** Pancreatic ductal adenocarcinoma, mRNA binding, Motility, Invasion, Adhesion

## Abstract

**Background:**

Over-expression of insulin-like growth factor 2 mRNA binding protein 3 (IMP3) is correlated with poor prognosis in pancreatic ductal adenocarcinoma (PDAC). Previous studies examining other cancer types have implicated IMP3 in the regulation of several cellular functions that are characteristic of tumour cells. However, the role of this oncofetal protein in PDAC progression remained unclear.

**Methods:**

Using siRNA, we examined the effect of IMP3 inhibition on the motility, invasive ability, and matrix adhesion of PDAC cells. In addition, we also evaluated the expression of cytoskeleton-associated genes following IMP depletion.

**Results:**

Knockdown of IMP3 significantly decreased the motility, invasion, and extracellular matrix adhesion of select PDAC cells *in vitro*. In addition, IMP3-depleted cells exhibited lower levels of CD44 protein and KIF11 mRNA. Moreover, we also observed a reduction in downstream RhoA signaling following IMP3 knockdown, indicating that IMP3 modulates the levels of proteins involved in cytoskeletal organization.

**Conclusions:**

These results suggest that IMP3 facilitates PDAC progression by enhancing the pro-metastatic behaviour of tumour cells.

**Electronic supplementary material:**

The online version of this article (doi:10.1186/s12885-015-1251-8) contains supplementary material, which is available to authorized users.

## Background

Pancreatic ductal adenocarcinoma (PDAC) is one of the most lethal malignancies with a 5-year survival rate of 1-4% and a median survival of 3–6 months [1]. The poor prognosis of PDAC has been attributed to advanced disease at presentation, limited impact of conventional chemotherapies on disease progression, and subsequent metastatic spread and disease recurrence [2,3]. The shortage of therapeutic options for PDAC underscores the need for molecularly targeted agents that can improve clinical outcomes.

Insulin-like growth factor-2 (IGF-2) mRNA binding protein 3 (IMP3) is an oncofetal protein that may be involved in the malignancy of PDAC. Over-expression of IMP3 in PDAC tissues relative to non-malignant pancreatic tissue is well-documented [4-6]. Interestingly, we have found that IMP3 expression was highest in poorly differentiated, grade 3 tumours [6]. Notably, the results of our study indicate that IMP3 expression is an independent predictor of overall survival and is correlated with poor patient prognosis [6]. However, it is unclear whether IMP3 plays an active role in facilitating PDAC progression.

Binding of IMP3 to mRNA transcripts exerts post-transcriptional control that influences key cellular functions involved in cancer progression. Loss-of-function experiments indicate that IMP3 is involved in the regulation of proliferation, motility, and invasion of leukemic [7], cervical carcinoma [8], glioblastoma [9], and oral carcinoma cells [10]. Hence, we hypothesize that IMP3 may be playing a similar role in PDAC.

The objective of this study was to determine the influence of IMP3 on the phenotype of PDAC cells. Using siRNA-mediated inhibition, the current study demonstrated that knockdown of IMP3 significantly reduced migration, invasion, and adhesion of pancreatic cancer cells. Subsequently, the effect of IMP3 inhibition on the expression of key proteins involved in adhesion and cytoskeletal organization was examined. Our results showed that IMP3 regulates the expression of CD44 and KIF11, independent of AKT, ERK-1/2, and FAK signaling. Thus, IMP3 inhibition may provide an avenue towards delaying the progression of PDAC.

## Methods

### Cell culture

Human pancreatic ductal epithelial (HPDE)-mock and KRASV12-transformed HPDE cells (a gift from Dr. Ming-Sound Tsao, University of Toronto, Canada) were maintained in serum-free keratinocyte medium (Invitrogen). HPAFII, MiaPaCa-2, PANC-1, and Hs766T, were obtained from American Type Culture Collection and cultured in the growth media recommended by ATCC. L3.6pl cells (a gift from Dr. Isiah J. Fidler, MD Anderson Cancer Center, Houston, TX) were cultured in MEM supplemented with 10% fetal bovine serum, 2% vitamins, 200 mM L-glutamine, 100 mM sodium pyruvate, and 1% non-essential amino acids. Cells were maintained at 37°C with 5% CO_2_ and passaged regularly at 70-80% confluence to ensure growth in the exponential phase.

Short interfering RNA transfection. The human IMP3 ON-TARGETplus SMARTpool siRNA (Thermo Fisher Scientific) contains a mixture of four siRNA which targets distinct coding region sequences of IMP3 (NM_006547). ON-TARGETplus non-targeting pool (Thermo Fisher Scientific) was used as the scrambled control. One day prior to transfection, the cells were seeded to ensure that density was at 40-50% confluence at the time of transfection. Scrambled or IMP3 siRNA were transfected into Hs766T and PANC-1 (200 nM) and L3.6pl (50 nM) using Lipofectamine RNAiMAX (Invitrogen) according to the manufacturer’s instructions. Forty-eight hours after transfection, the cells were harvested for functional studies or molecular analyses as described below. The individual siRNAs comprising the pooled siRNA solution were used in conjunction with Hs766T cells and reduced the motility of the cells. A control scrambled siRNA sequence was used to asses baseline motility. The results are shown in Additional file 1: Figure S1.

Cell migration and invasion assays. BioCoat matrigel-coated chambers and BioCoat control inserts (BD Biosciences) were used to assess migration and motility, respectively. A total of 2.5 × 104 cells were suspended in serum-free DMEM and added onto the upper chamber. DMEM with 10% FBS added to the lower chamber served as chemoattractant. After 22 h of incubation at 37°C and 5% CO2, cells that have invaded and migrated through the chambers were fixed in formalin and stained with H&E for visualization. All cells on the invasion inserts and 12 selected fields on the migration inserts were counted using bright field microscopy at 10X (Axiovert40C, Zeiss). Three replicate inserts were performed for each experiment, and the experiments were repeated 3 times.

### Scratch wound healing assay

The motility of L3.6pl cells were assessed using scratch wound healing assays. Forty-eight hours after siRNA transfection, plates were scratched linearly using a 200 μl pipette tip. Cells were washed with PBS and cultured in supplemented MEM. Phase-contrast images were captured at 3 different sections along the scratch at baseline (T0) and 24 h (T24) after wounding using Axiovert40C (Zeiss) at 20X. The area of the scratch was quantified using ImageJ, and wound coverage was calculated as the difference in areas between T0 and T24.

### Adhesion assay

Forty-eight hours after siRNA transfection, the cells were detached with 0.25% trypsin-EDTA (Invitrogen). After washing with PBS, 1.0 × 10^6^ cells were then seeded onto the extracellular matrix (ECM) adhesion array (Millipore). The assay was performed in accordance with the manufacturer’s instructions.

### ELISAs

Total cellular protein was collected 48 h after siRNA transfection. The levels of GTP-bound RhoA, IGF-2, and NGFβ in the protein lysates were quantified with RhoA G-LISA (Cytoskeleton), non-extraction IGF-2 ELISA (Diagnostic Systems), and NGF Emax ImmunoAssay Systems (Promega), respectively. The assays were performed according to the manufacturer’s recommended protocol.

### Western blot analysis

Cells were lysed in buffer containing protease inhibitors [50 mmol/L HEPES (pH 8.0), 10% glycerol, 1% Triton X-100, 150 mmol/L NaCl, 1 mmol/L EDTA, 1.5 mmol/L MgCl2, 100 mmol/L NaF, and 10 mmol/L Na_4_P_2_O_7_H_2_O supplemented with 5 μg/mL leupeptin, 5 μg/mL aprotinin, 100 μg/mL phenylmethylsulfonylfluoride and 37 μg/mL Na_3_VO_4_]. Protein concentrations were quantified using Micro BCA Protein Assay (Thermo Scientific). Total cellular protein was heat-denatured, resolved on 12% SDS-PAGE, and transferred onto nitrocellulose membrane. Membranes were blocked in 5% skim milk for 1 h at room temperature followed by an overnight incubation at 4°C with primary antibodies against IMP3 (1:1000; M3626 Dako), CD44 (1:800; ab119863 Abcam), RhoA (1:1000; Cytoskeleton), phospho-FAK Y397 (1:1000 ab4803 abcam), phospho-AKT S473 (1:1000; 3787S Cell Signaling Technology), and phospho-Erk1/2 T202/Y204 (1:1000; 9101S Cell Signaling Technology). Membranes were probed with horseradish peroxidase-conjugated goat anti-mouse IgG, goat anti-rabbit IgG (1:5000; Promega), or goat anti-rat IgG (abcam) for 1 h at room temperature followed by detection with SuperSignal West Pico Chemiluminescent Substrate (Thermo Scientific) and imaging with ChemiDoc MP (Bio-Rad). Membranes were then stipped and re-probed for β-actin (1:5000; ab8227 Abcam), total FAK (1:1000; ab40794 Abcam), total Akt (1:1000; 9272 Cell Signaling Technology), or total Erk1/2 (1:1000; 9102 Cell Signaling Technology). Band densities were quantified using Image Lab (Bio-Rad).

### Messenger ribonucleotide immunoprecipitation assay

IMP3 and associated mRNAs were isolated from cell lysates through immunoprecipitation. Intracellular proteins were collected by incubating cells in polysome lysis buffer. The lysates were pre-cleared by adding non-immune rabbit IgG (20 μg) for 1 h at 4°C followed by incubation with 50 μl of Protein G-agarose beads (Sigma-Aldrich) suspended in NT2 buffer supplemented with 5% BSA. The protein concentrations of the pre-cleared lysates were determined using BCA assay (Thermo Scientific). To precipitate IMP3, 1.5 mg of protein was incubated overnight with Protein G-agarose beads coated with 40 μg of rabbit anti-human IMP3 (MBL Intl) or normal rabbit IgG (Sigma-Aldrich) resuspended in NT2 buffer supplemented with RNase Out (Invitrogen), VRC, leupeptin, aprotinin, PMSF, and sodium orthovanadate. After incubation at room temperature for 3 h, the beads were collected by centrifugation, washed with NT2 buffer, and incubated with 20 units of DNase I (Qiagen) in 100 μl of NT2 buffer for 20 minutes at 30°C. After washing with NT2 buffer, the beads were pelleted by pulse centrifugation and resuspended in NT2 buffer supplemented with 30 μg of protease K (Sigma-Aldrich) and 0.1% SDS for 30 min at 55°C. RNA was extracted using Trizol (Invitrogen) following the manufacturer’s protocol.

### Quantitative real-time reverse transcriptase-polymerase chain reaction (qRT-PCR)

The transcription of kinesin KIF11, KIF14, KIF23, IGF-2, NGFβ, and GAPDH were measured using qRT-PCR. First-strand cDNA was synthesized from 1 μg of cellular RNA extracted using RNeasy Plus Mini kit (Qiagen) with on-column DNA digestion or 11 μl of RNA collected from RIP assay using Oligo(dT)20 primers (Invitrogen) and SuperScript III (Invitrogen) following the manufacturer’s recommended protocol. qRT-PCR was performed using primers listed in the Table 1, and amplification was monitored using SYBR Green. The cycling parameters included an initial denaturation at 50°C for 30 min, followed by 95°C for 15 min, and 50 cycles of annealing and extension at 94°C for 20 s and 60°C for 1 min. Under these conditions, the amplification efficiencies of the targets were shown to be comparable to that of the endogenous control, GAPDH. Fold difference was analyzed using 2^-ΔΔC^_T._

**Table 1:** **Primer sequences**

	Forward sequence (5′→3′)	Reverse sequence (5′→3′)	Source
**IGF-II**	AAGTCGATGCTGGTGCTTCT	CGGAAACAGCACTCCTCAA	[7]
**NGF**β	ATACAGGCGGAACCACACTC	TGCTCCTGTGAGTCCTGTTG	[37]
**KIF11**	CAGCTGAAAAGGAAACAGCC	ATGAACAATCCACACCAGCA	[38]
**KIF14**	TTGCTACGATTAGTCCCGCT	GCTTTGCAATTTCTGCCTTC	[38]
**GAPDH**	TTTAACTCTGGTAAAGTGGATATTGTTG	ATTTCCATTGATGACAAGCTTCC	[7]

### Statistical analyses

All results were presented as mean ± SEM. Statistical analyses were carried out with repeated measures analysis of variance (ANOVA), followed by the Dunnett post-hoc test, with P < 0.05 as the criterion for statistical significance. Data were presented as means of at least 3 independent experiments.

## Results

### Expression of IMP3 in pancreatic cancer cell lines

**Figure 1:**
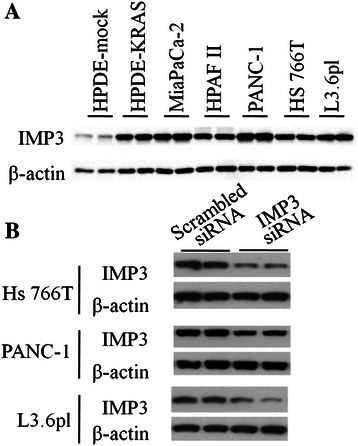
Expression of IMP3 in PDAC cell lines. **(A)** Western blots showing basal expression of IMP3 protein in mock-transfected human pancreatic ductal epithelial cells (HPDE-mock), KrasV12-transfected HPDEs (HPDE-KRAS), and several pancreatic cancer cell lines. **(B)** Treatment with IMP3 siRNA reduced the IMP3 levels in Hs766T, PANC-1, and L3.6pl. Scrambled siRNA-transfected counterparts were included as controls.

The expression of IMP3 protein in pancreatic cancer cell lines derived from primary tumours (PANC-1 and MiaPaCa-2) and distant metastatic (HPAF-II, Hs766T, L3.6pl) sites is shown in Figure 1A. IMP3 was highly expressed in human pancreatic cancer cell lines and interestingly, in KRAS^V12^-transformed human pancreatic ductal epithelial cells as well. In contrast, human pancreatic ductal epithelial cells (HPDE-mock) express markedly lower levels of IMP3.

### IMP3 knockdown decreases motility, invasion, and matrix adhesion

To examine the influence of IMP3 on cellular behaviour, the levels of IMP3 in pancreatic cancer cell lines were depleted with RNA interference. Relative to scrambled siRNA-transfected controls, treatment with human IMP3 SMARTpool siRNA duplexes for 48 h achieved significant reductions of IMP3 levels in Hs766T (46%), PANC-1 (45%), and L3.6pl (58%) (Figure 1B) without affecting proliferation.

**Figure 2:**
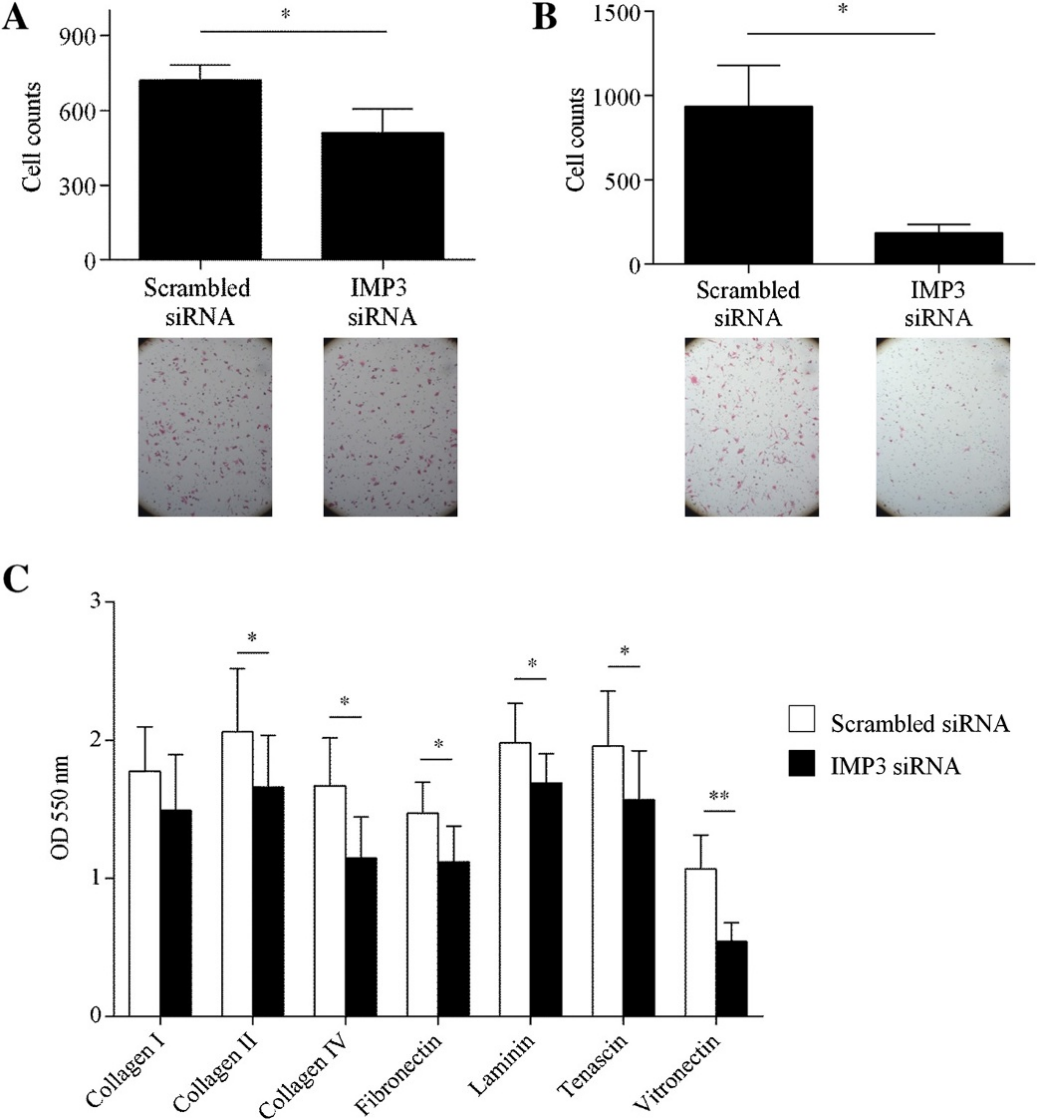
Effect of IMP3 knockdown on motility, invasion, and matrix adhesion of Hs766T. **(A)** The motility of Hs766T in Boyden chambers was significantly decreased following siRNA-mediated inhibition of IMP3. *Inset* Representative images (10X) of motile Hs766T 24 h after seeding. **(B)** The invasive potential of Hs766T was evaluated using Matrigel-coated Boyden chambers. IMP3 depletion resulted in a significant decrease in the invasive potential of Hs766T. *Inset* Representative images (10X) of invasive Hs766T 24 h after seeding. **(C)** Adhesion to collagen I (Coll I), collagen II (Coll II), collagen IV (coll IV), fibronectin (FN), tenascin (TN), laminin (LN), tenascin (TN), and vitronectin (VN) was quantified spectrophotometrically. Absorbance at 550 nm is proportional to the number of adherent cells. *P < 0.05, **P < 0.01 relative to scrambled siRNA-transfected controls.

Depletion of IMP3 led to a significant decrease in the motility of Hs766T, a PDAC cell line derived from a lymphatic metastasis (Figure 2A). However, knocking down IMP3 did not affect the movement of PANC-1 through the transwell or the ability of L3.6pl cells to cover a scratch on the culture plate (Additional file 2: Figure S2).

Using modified Boyden chamber assays, we examined whether IMP3 is involved in regulating the ability of cells to penetrate tissue barriers *in vitro*. As shown in Figure 2B, IMP3 depletion resulted in a 4-fold decrease in the ability of Hs766T to invade the basement membrane. In contrast, the invasive ability of PANC-1 was not significantly affected by IMP3 depletion (Additional file 2: Figure S2). The effect of IMP3 inhibition on L3.6pl cell invasion could not be determined with this assay as the cells did not penetrate the matrix.

Next, we assessed the effect of IMP3 depletion on the adhesion of pancreatic cancer cells to proteins in the extracellular matrix (ECM). In Hs766T, the inhibition of IMP3 led to marked reductions in cellular adhesion to ECM proteins including collagen IV, fibronectin, laminin, tenascin, and fibronectin but not to collagen I and collagen II (Figure 2C). We did not observe significant changes in the adhesion of PANC-1 and L3.6pl to ECM proteins (Additional file 3: Figure S3).

### IMP3 is involved in the regulation of genes involved in cell migration

**Figure 3:**
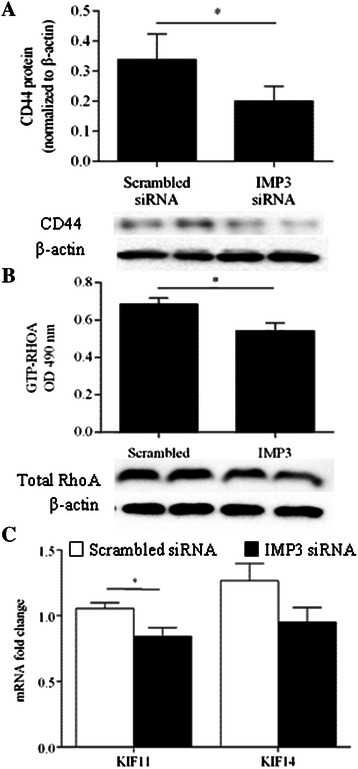
IMP3 regulates CD44 and KIF11. **(A)** Total CD44 protein was measured in whole cell lysates 48 h after transfection. Treatment with IMP3 siRNA resulted in a significant decrease in CD44 protein relative to scrambled siRNA-treated controls. *Inset* Representative blots of CD44 and corresponding β-actin. *P < 0.05 relative to scrambled siRNA-transfected controls. **(B)** Levels of KIF11 and KIF14 mRNA were measured 48 h after siRNA transfection and expressed in amount of fold-change relative to scrambled siRNA-treated controls. *P < 0.05 relative to scrambled siRNA-transfected controls.

Based on earlier reports of interactions between IMP3 and mRNAs that contribute to the migration of other cancer cell lines [8,9], we decided to assess the effect of knocking down IMP3 on the expression of receptors for ECM proteins and microtubule-associated motor proteins. Knockdown of IMP3 in Hs766T cells resulted in a significant decrease in the levels of CD44 protein (Figure 3A) and active, GTP-bound RhoA but not total RhoA (Figure 3B). In contrast, we did not observe significant changes in the expression of β1 integrin and levels of total and phosphorylated FAK between IMP3-depleted cells and controls (Additional file 4: Figure S4). To assess the expression of motor proteins following IMP3 knockdown, we quantified the mRNA levels of kinesins implicated in PDAC cell motility and invasion. Results of qRT-PCR revealed that knocking down IMP3 knockdown significantly reduced the expression of kinesin KIF11 but not kinesin KIF14 (Figure 3C).

### Effect of IMP3 is independent of IGF-2 and NGFβ

**Figure 4:**
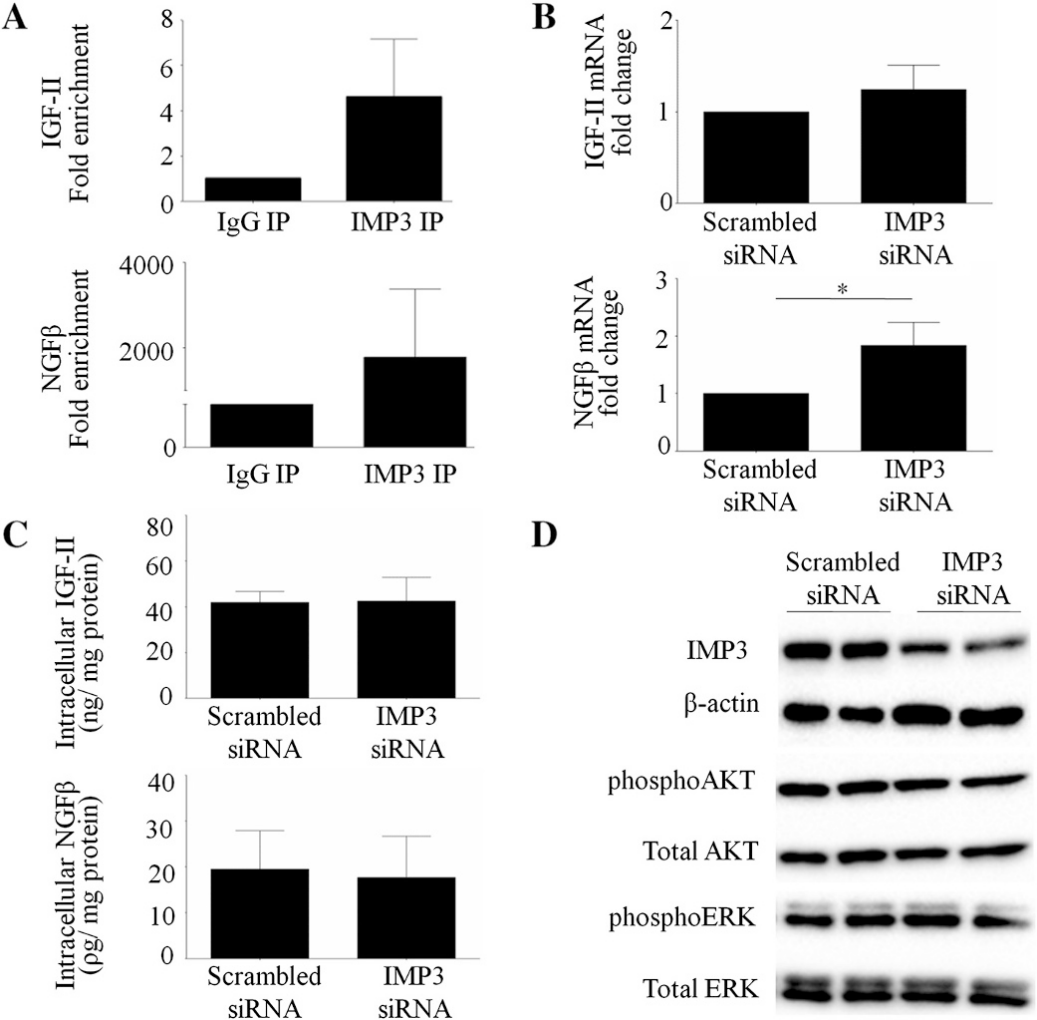
Effect of IMP3 is independent of IGF-2 and NGFβ. Cell lysates were subjected to immunoprecipitation using anti-human IMP3 and rabbit IgG. RNA extracted from the precipitates were analyzed using qRT-PCR. **(A)** Higher amplifications of IGF-2 mRNA and NGFβ mRNA were detected in IMP3 pull-down fraction relative to IgG. **(B)** Treatment with IMP3 siRNA did not alter the mRNA levels of IGF-2 while it increased NGFβ mRNA relative to scrambled siRNA-treated counterparts. qRT-PCR was used to measure mRNA levels following siRNA transfection. **(C)** Reduction in IMP3 did not alter levels of IGF-2 and NGFβ protein in Hs766T. Intracellular protein levels were measured using ELISAs. **(D)** Knocking down IMP3 did not alter the levels of phosphorylated and total ERK as well as phosphorylated and total AKT.

IMP3 has previously been shown to facilitate the translation of IGF-2 mRNA [7,9] and increase the levels of NGFβ in pancreatic ductal cells [11]. Hence, we first examined whether facilitation of growth factor signaling mediates the influence of IMP3 on the phenotype of Hs766T cells. The results of ribonucleoprotein immunoprecipitation assays showed an enrichment of IGF-2 and NGFβ mRNAs in the IMP3 pull-down fraction (Figure 4A), indicating that IMP3 interacts with these sequences.

To determine whether the deactivation of an IGF-2 or NGFβ dependent pathway underlies the observed effects of IMP3 depletion on the migratory behaviour of Hs766T, we measured the expression and translation of these growth factors and their associated signaling cascades following IMP3 knockdown. As shown in Figure 4B, there was no significant difference in IGF-2 mRNA levels between IMP3-depleted cells and scrambled siRNA-treated controls. Interestingly, knockdown of IMP3 resulted in a 3-fold increase in the levels of NGFβ mRNA. However, results of our ELISAs indicate that IMP3 knockdown did not alter the intracellular protein levels of IGF-2 and NGFβ. (Figure 4C). Moreover, we did not observe changes in the levels of total and phosphorylated AKT and ERK (Figure 4D).

## Discussion

Over-expression of IMP3 has previously been reported in PDAC. However, the contribution of this oncofetal protein to disease progression has not yet been clearly defined. In this study, we demonstrated that knockdown of IMP3 impedes motility, invasion, and matrix adhesion of pancreatic cancer cells. Furthermore, siRNA-mediated inhibition of IMP3 reduced the levels of CD44 protein, KIF11 mRNA, and RhoA activation, suggesting that the effect of IMP3 on facilitating metastatic potential is likely associated with alterations in cytoskeletal dynamics. It is noteworthy that in PDAC cells, knockdown of IMP3 did not alter the activation of canonical signal transduction pathways associated with cell proliferation and movement including AKT, ERK-1/2, and FAK. Thus, IMP3 inhibition presents an alternative means of selectively impeding cell migration to potentially retard the metastatic potential of PDAC.

IMP3 is an mRNA-binding protein shown to be over-expressed in PDAC and various other malignancies including cervical [8], endometrial [12,13], bladder [14], lung [15], renal cell [16,17] and breast carcinomas [18,19] as well as glioblastoma [9] and malignant melanoma [20]. The re-expression of IMP3 in KRAS^V12^-transformed cells as well as in cells harboring an activating K-ras mutation indicates that IMP3 induction may be concomitant with acquisition of K-ras mutations. Recently, epidermal growth factor receptor (EGFR) signaling has been shown to regulate IMP3 expression. In both oral squamous cell carcinoma [21] and breast carcinoma cells [19], pharmacological inhibition of EGFR resulted in decreased expression of IMP3. Given that EGFR over-expression has previously been identified in PDAC [22], it is plausible that enhanced EGFR signaling may also be influencing IMP3 expression. The mechanisms enabling IMP3 re-expression in PDAC remains to be elucidated.

While IMP3 inhibition led to significant impairment in the behavior of Hs766T, this effect was not observed in PANC-1 and L3.6pl. Recent analysis of gene expression patterns revealed that a sub-set of genes involved in cellular adhesion and motility are differentially expressed in PDAC cell lines [23]. For instance, while mutations in K-ras, P16, and P53 have been identified in Hs766T and PANC-1, DPC4/Smad4 inactivation has only been reported in Hs766T and not in PANC-1 [24] and L3.6pl [25]. Interestingly, decreased DPC4/Smad4 signaling has been shown to enhance PDAC cell motility and invasion [26]. In addition, loss of DPC4/Smad4 has also been associated with PDAC progression [27,28]. Given the results of our study, it is likely that the role of IMP3 in facilitating metastatic potential is more pronounced in DPC4/Smad4-negative tumour cells. Further investigation into mechanisms underlying the observed differences in phenotypic response to IMP3 depletion is warranted, as it may uncover biomarkers that can predict response to pharmacologic agents that target IMP3.

The observed decrease in motility, invasion, and matrix adhesion of Hs766T following IMP3 knockdown suggests that IMP3 facilitates the pro-metastatic behavior of a sub-set of pancreatic cancer cells. This role of IMP3 in pancreatic cell movement is consistent with reports obtained in other cell lines. Previous studies have shown that IMP3 is crucial for maintaining the invasive phenotype of cervical carcinoma [8], oral squamous cell carcinoma [21], hepatocellular carcinoma [29], and glioblastoma cells [9]. Furthermore, over-expression of IMP3 *in vivo* has been shown to induce acinar-ductal metaplasia [11] and increase the formation of malignant tumours in a lung model of metastasis [9]. Unfortunately, at the present time a lack of suitable pancreatic tumor models for studying metastasis preclude extension of our studies in the *in vivo* setting. More importantly, we have previously established a correlation between IMP3 expression and patient prognosis in PDAC [6]. Consistent with these findings, the results of our current study support the notion that IMP3 enhances the aggressiveness of PDAC by promoting cancer cell dissemination.

Besides interacting with IGF-2 mRNA, IMP3 has been shown to bind to and regulate the translation of multiple mRNA sequences [30]. In HeLa cells, Vikesaa *et al*. have previously shown that IMP3 binds to CD44 mRNA [8]. As an adhesion molecule, CD44 interacts with ECM proteins including hyaluronan, collagen, fibronectin, and laminin [31-34]. In Hs766T, we have found that IMP3 knockdown resulted in a marked decrease in CD44 protein. Coupled with observations of decreased matrix adhesion in IMP3-depleted cells, our results suggest that IMP3 is involved in regulating the levels of CD44 protein in PDAC cells. In breast cancer cells, CD44 has been shown to stimulate the guanine exchange activity of p115RhoGEF leading to activation of RhoA, a GTPase involved in cytoskeletal organization and adhesion. In PDAC cells, we demonstrated that knocking down IMP3 resulted in lower levels of active, GTP-bound RhoA. Thus, the observed impairment of pancreatic cancer cell behavior following IMP3depletion is likely due to inhibition of CD44-RhoA signaling.

In addition to CD44, our results also indicate that IMP3 regulates KIF11 mRNA. Over-expressed in PDAC cell lines [35,36] and in pancreatic tumours (unpublished data), KIF11 is a mitotic kinesin that has been shown to promote cancer cell proliferation and tumour formation [35]. More recently, inhibition of KIF11 has been reported to decrease the migration and invasion of PDAC cells without affecting cell proliferation [36], suggesting that KIF11 also plays a role in coordinating cell movement. Taken together, our results showed that IMP3 expression promotes matrix adhesion, motility and invasion of pancreatic cancer cells by enhancing CD44 and KIF11 expression. Profiling and pathway analysis of genes associated with cytoskeletal organization, motility, and ECM interaction following IMP3 knockdown in PDAC cells would be instrumental in identifying additional molecules that promote the metastatic spread of PDAC.

## Conclusions

Our results demonstrate that IMP3 is involved in facilitating the pro-metastatic behavior of a subset of pancreatic cancer cells. This effect is likely due to increased translation of mRNAs that contribute to motility, invasion and matrix adhesion including CD44 and KIF11. Given the poor efficacy of currently available treatments in PDAC, pharmacologic inhibitors of IMP3 may represent a viable therapeutic strategy by altering pancreatic cancer cell behavior and halting/delaying pancreatic tumour metastasis.

## Additional files

Additional file 1: Figure S1. Effect of IMP3 on motility of Hs766T. Cells transfected with different siRNA sequences targeting IMP3 or scrambled siRNA were washed and resuspended in serum-free DMEM. Cells were then deposited on the upper chamber of 0.8 μm PET wells (BD). The lower compartment were filled with DMEM supplemented with 10% FBS. Cells that have traveresed the membrane were fixed and stained after 22 hours. Cells in 12 different fields were counted from 3 different chambers for each treatment. Bars represent average number of motile cells ± SEM, n = 2.
Additional file 2: Figure S2. Effect of IMP3 knockdown on cell motility and invasion. (A) IMP3 depletion did not affect the movement of Panc1 through transwell chambers. (B) Knocking down IMP3 resulted in a slight decrease in the ability of L3.6pl to cover a scratch on the culture plate. However, this trend was not found to be statistically significant. (C) Deceasing IMP3 levels did not significantly alter the invasive ability of Panc1.
Additional file 3: Figure S3. Effect of IMP3 knockdown on cellular adhesion to extracellular matrix proteins. Decreasing levels of IMP3 did not significantly alter the ability of Panc1 (A) and L3.6pl (B) to adhere to extracellular matrix proteins. Bovine serum albumin (BSA)-coated wells were included as negative controls. Adhesion was quantified spectrophotometrically, and absorbance at 550 nm is proportional to the number of adherent cells.
Additional file 4: Figure S4. Effect of IMP3 knockdown on β1 integrin signaling in Hs766T. (A) Representative western blot of β1 integrin and β-actin in IMP3-depleted cells and scrambled siRNA-treated control. Expression of β1 integrin was found to be similar between IMP3-depleted cells and scrambled siRNA-treated controls. (B) Representative western blot of phosphorylated FAK and total FAK in IMP3 depleted cells and controls. Levels of phosphorylated and total FAK were comparable between conditions.

## Abbreviations

PDAC: Pancreatic ductal adenocarcinoma
IMP3: Insulin-like growth factor 2 mRNA binding protein 3
IGF2: Insulin growth factor 2
MMP9: Matrix metalloproteinase 9
FAK: Focal adhesion kinase
HPDE: Human pancreatic ductal epithelial
EGFR: Epidermal growth factor receptor
