# Supplementary material for: The involvement of insulin-like growth factor 2 binding protein 3 (IMP3) in pancreatic cancer cell migration, invasion, and adhesion
Source: BMC Cancer. 2015 Apr 11;15:266. doi: 10.1186/s12885-015-1251-8 (PMC4403680; doi:10.1186/s12885-015-1251-8)
Supplement: Additional file 1: Figure S1. — Effect of IMP3 on motility of Hs766T. Cells transfected with different siRNA sequences targeting IMP3 or scrambled siRNA were washed and resuspended in serum-free DMEM. Cells were then deposited on the upper chamber of 0.8 μm PET wells (BD). The lower compartment were filled with DMEM supplemented with 10% FBS. Cells that have traveresed the membrane were fixed and stained after 22 hours. Cells in 12 different fields were counted from 3 different chambers for each treatment. Bars represent average number of motile cells ± SEM, n = 2. [file 12885_2015_1251_MOESM1_ESM.pdf]

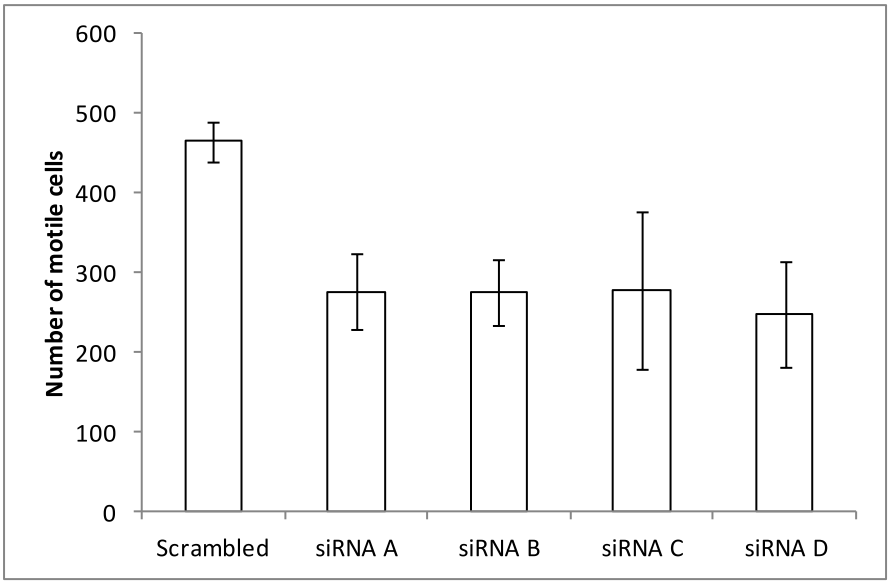

siRNA sequences
siRNA A: CGUCCAAGAUCAAGCGGCA
siRNA B: GGGAGGACUACACGCGCUA
siRNA C: UUUUAUAAACUGAGCGAGA
siRNA D: CUGGAAGCCUAGCGGAUCU

Additional Figure 4 S4: Effect of IMP3 on motility of Hs766T. Cells transfected with different siRNA sequences targeting IMP3 or scrambled siRNA were washed and resuspended in serum-free DMEM. Cells were then deposited on the upper chamber of 0.8µm PET wells (BD). The lower compartment were filled with DMEM supplemented with 10% FBS. Cells that have traveresed the membrane were fixed and stained after 22 hours. Cells in 12 different fields were counted from 3 different chambers for each treatment. Bars represent average number of motile cells ± SD, n=2
