# Supplementary figures and images for: The involvement of insulin-like growth factor 2 binding protein 3 (IMP3) in pancreatic cancer cell migration, invasion, and adhesion
Source: BMC Cancer. 2015 Apr 11;15:266. doi: 10.1186/s12885-015-1251-8 (PMC4403680; doi:10.1186/s12885-015-1251-8)

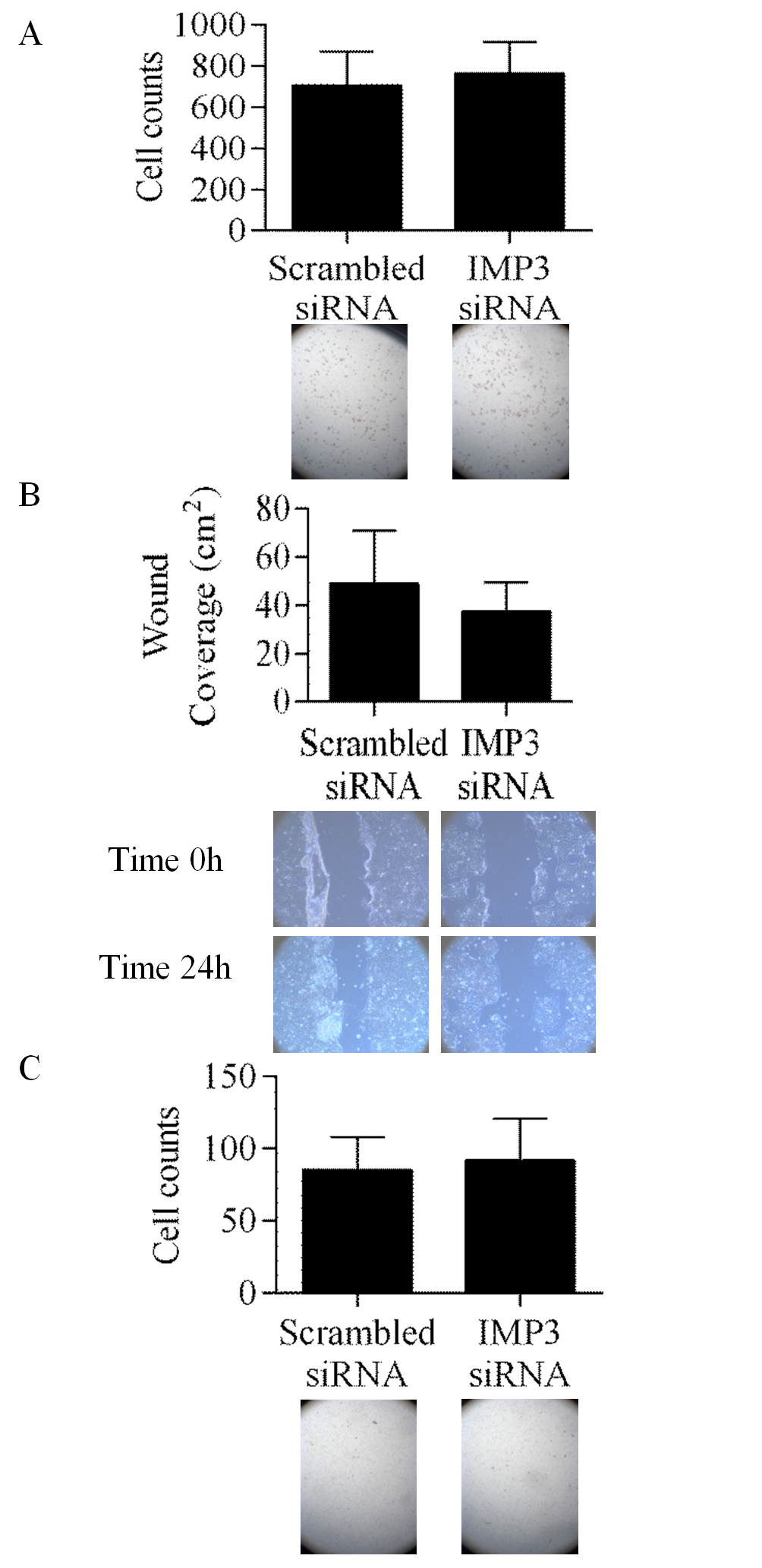

Supplement: Additional file 2: Figure S2. — Effect of IMP3 knockdown on cell motility and invasion. (A) IMP3 depletion did not affect the movement of Panc1 through transwell chambers. (B) Knocking down IMP3 resulted in a slight decrease in the ability of L3.6pl to cover a scratch on the culture plate. However, this trend was not found to be statistically significant. (C) Deceasing IMP3 levels did not significantly alter the invasive ability of Panc1. [file 12885_2015_1251_MOESM2_ESM.tif]

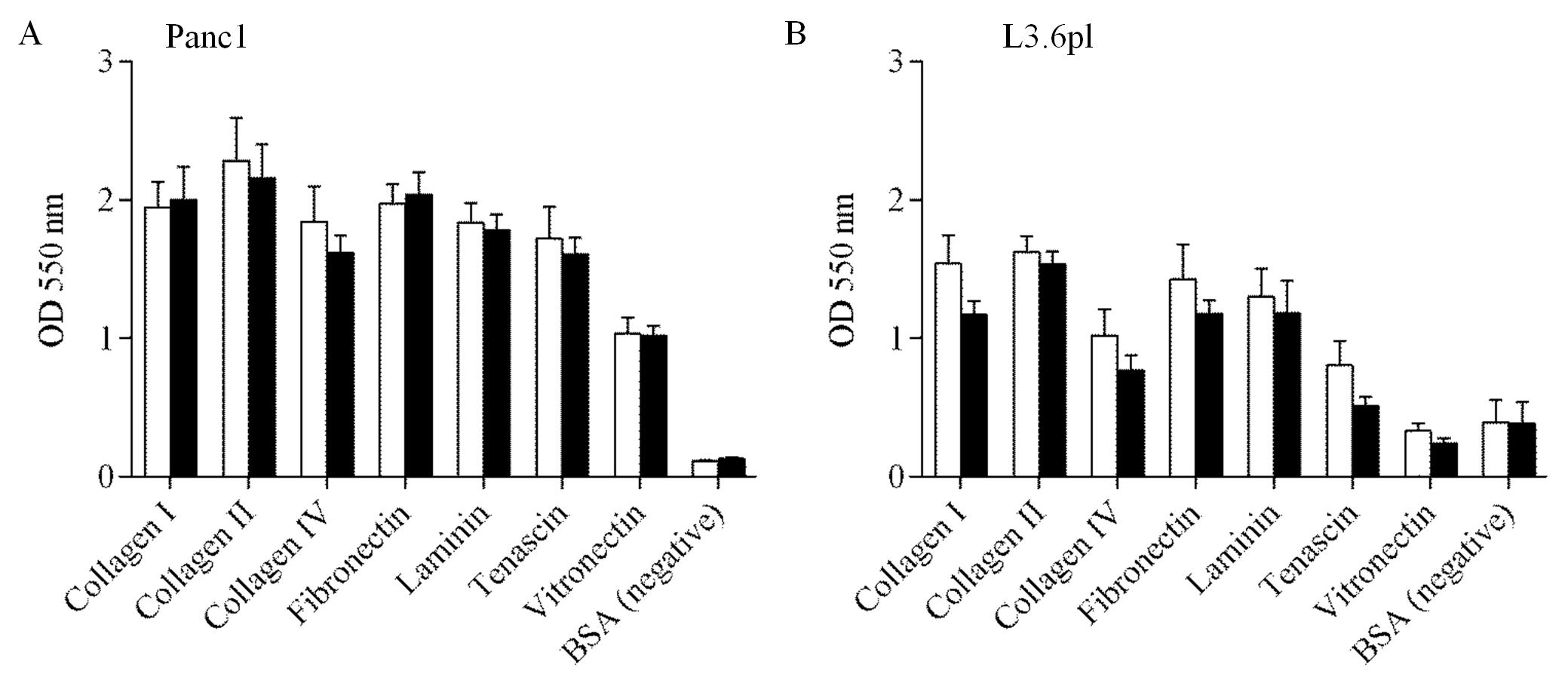

Supplement: Additional file 3: Figure S3. — Effect of IMP3 knockdown on cellular adhesion to extracellular matrix proteins. Decreasing levels of IMP3 did not significantly alter the ability of Panc1 (A) and L3.6pl (B) to adhere to extracellular matrix proteins. Bovine serum albumin (BSA)-coated wells were included as negative controls. Adhesion was quantified spectrophotometrically, and absorbance at 550 nm is proportional to the number of adherent cells. [file 12885_2015_1251_MOESM3_ESM.tif]

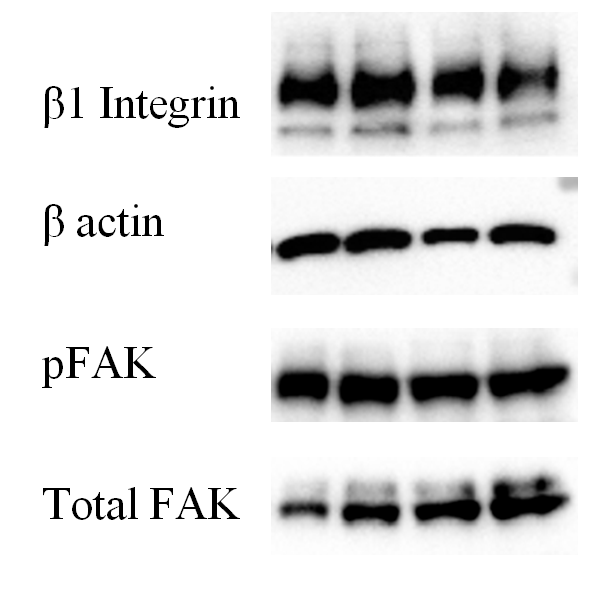

Supplement: Additional file 4: Figure S4. — Effect of IMP3 knockdown on β1 integrin signaling in Hs766T. (A) Representative western blot of β1 integrin and β-actin in IMP3-depleted cells and scrambled siRNA-treated control. Expression of β1 integrin was found to be similar between IMP3-depleted cells and scrambled siRNA-treated controls. (B) Representative western blot of phosphorylated FAK and total FAK in IMP3 depleted cells and controls. Levels of phosphorylated and total FAK were comparable between conditions. [file 12885_2015_1251_MOESM4_ESM.tif]
